# *Ac*MNPV Core Gene *ac109* Is Required for Budded Virion Transport to the Nucleus and for Occlusion of Viral Progeny

**DOI:** 10.1371/journal.pone.0046146

**Published:** 2012-09-26

**Authors:** Victoria Alfonso, Guillermo A. Maroniche, Sol R. Reca, María Gabriela López, Mariana del Vas, Oscar Taboga

**Affiliations:** 1 Instituto de Biotecnología, CICVyA, Instituto Nacional de Tecnología Agropecuaria (IB-INTA), Hurlingham, Buenos Aires, Argentina; 2 Instituto de Microbiología y Zoología Agrícola, CICVyA, Instituto Nacional de Tecnología Agropecuaria (IMyZA-INTA), Hurlingham, Argentina; Lady Davis Institute for Medical Research, Canada

## Abstract

The *Autographa californica* multiple nucleopolyhedrovirus (*Ac*MNPV) *ac109* core gene has been previously characterized as an essential late gene. Our results showed that budded virions could be detected in supernatants of infected Sf-9 cells, even when *ac109* knockout viruses displayed a single-cell infection phenotype. Moreover, confocal microscopy analysis revealed that budded virions can enter the cytoplasm but are unable to enter the cell nucleus. This defect could be repaired by complementing *ac109* in *trans*. In addition, polyhedra of normal size could be detected in Sf-9 nuclei infected with *ac109* knockout viruses. However, electron microscopy demonstrated that these occlusion bodies were empty. Altogether, these results indicate that *ac109* is required for infectivity of both phenotypes of virus.

## Introduction

The *Baculoviridae* family is composed of a diverse group of viruses that infect mostly lepidopterans but also dipterans and hymenopterans [1]. All baculoviruses are enveloped viruses with a circular, double-stranded DNA genome ranging from 80 to 180 Kbp. The unique baculovirus life cycle is biphasic and complex. It displays two phenotypes of virions: budded virions (BVs) and occlusion-derived virions (ODVs) [2]. BVs are produced early in infection. These BVs are able to spread the infection horizontally to many cell types of larvae. In the last stages of the viral cycle, multiple nucleocapsids are enveloped in the cell nucleus and finally they are occluded in a proteinaceous matrix, termed polyhedra, to form ODVs. ODVs have evolved to resist desiccation and infect the epithelial cells once the polyhedra are dissolved in the alkaline environment of the lepidopteran midgut. BVs and ODVs share their nucleocapsids but differ in the origin and composition of their envelopes [3], [4].

The *Autographa californica* multiple nucleopolyhedrovirus (*Ac*MNPV) is the archetypical member of Alphabaculovirus, and is broadly used as a vector for the expression of recombinant proteins in insect cells and larvae [5]. Thirty one genes out of the 154 predicted open reading frames contained in *Ac*MNPV genome are conserved in all baculoviruses and they are referred as core genes [6]. Some of these core genes code for proteins of essential functions related to DNA replication, gene transcription, nucleocapsid structure and *per os* infectivity.

In particular, the *Ac*MNPV *ac109* core gene codes for a late structural protein essential for viral spread in cultured cell, but its participation in a defined step of the virus replication cycle has not been so far elucidated. Moreover, much controversy exists over most of the points studied. Lin et al. stated that Ac109 is required for nucleocapsid assembly and posterior BVs and ODVs production; in addition, these authors have pointed out that no occlusion body is formed in the absence of Ac109 [7]. Besides, Fang et al. have established that after transfection with an *ac109* deleted bacmid, non-infectious BVs and polyhedra are produced [8].

In this paper, we intended to elucidate the role of *ac109* in BV and ODV phenotypes. For this purpose, we analyzed the production and infectivity of both viral phenotypes resulting from an *ac109* knockout mutant. Confocal microscopy analysis showed that *ac109* knockout baculoviruses produced BVs that were unable to enter the nucleus of Sf-9 cells. Such capacity was restored by *trans* complementation of *ac109*. In addition, transmission electron microscopy (TEM) revealed that knockout viruses failed to occlude, even though polyhedra of normal size and appearance could be detected.

## Results

### Construction of an ac109 Knockout Baculovirus and its Phenotypic Characterization

**Figure 1:**
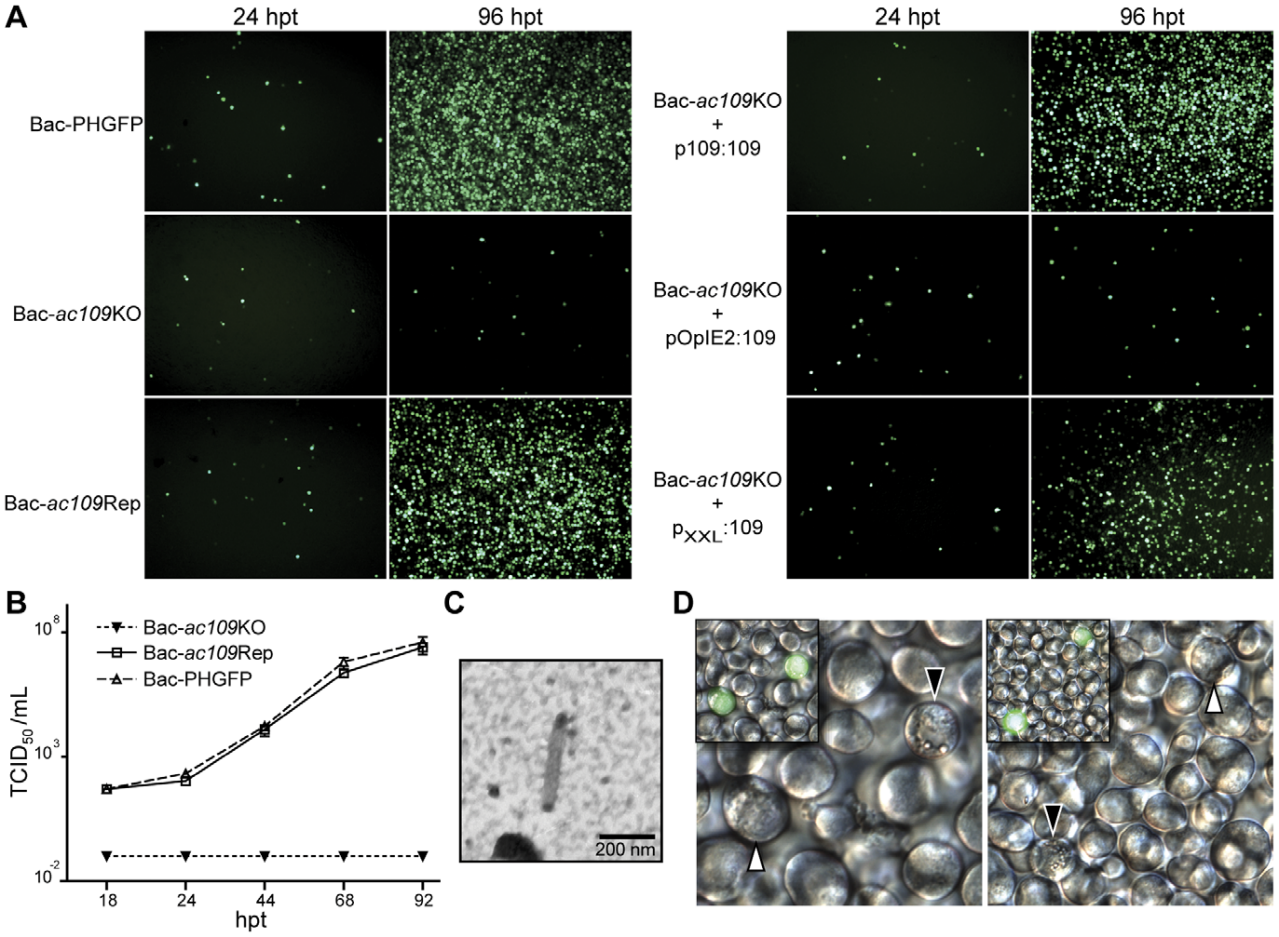
Characterization of Bac-*ac109*KO and complementation assays. (A) Fluorescence microscopy of Sf-9 cells transfected with DNA of Bac-PHGFP, Bac-*ac109*KO or Bac-*ac109*Rep bacmids (left panels) or cotransfected with Bac-*ac109*KO bacmid and one of the following plasmids: TOPO-p109-109, pIB109 and p_XXL_109 (right panels). eGFP fluorescence was monitored at 24 and 96 h post-transfection (hpt). Plasmid TOPO-p109-109 carried *ac109* driven by its own promoter (p109∶109), plasmid pIB109 harbored *ac109* under constitutive pOpIE2 promoter regulation (pOpIE2∶109) and plasmid p_XXL_109 carried *ac109* driven by a modified version of the very late polyhedrin promoter regulation (p_XXL_:109). (B) Production of infectious budded viruses (BVs). Sf-9 cells were transfected with Bac-*ac109*KO, Bac-PHGFP or Bac-*ac109*Rep bacmids, and at the indicated time points the supernatants were harvested, clarified and tittered by the end point dilution method. Titers were calculated as TCID_50_ per milliliter. Each sample was performed in triplicate. (C) Electronic microscopy of an *ac109*KO BV. Sf-9 cells were transfected with Bac-*ac109*KO bacmid, and 4 days post-transfection culture supernatant was harvested, clarified and concentrated by ultracentrifugation through a 25% w/v sucrose cushion. The pellet was adsorbed to copper grids, negatively stained and analyzed by transmission electronic microscopy. (D) Light (main figure) and fluorescence (left inner figure) microscopy of cells transfected with Bac-*ac109*KO bacmid. At 96 hpt, cells showing eGFP fluorescence were observed for polyhedra formation. White arrows indicate fluorescent cells without polyhedra and black arrows indicate fluorescent cells with polyhedra.

To determine the consequences of the lack of Ac109 in *Ac*MNPV life cycle, we constructed three bacmids (Bac-PHGFP, Bac-*ac109*KO and Bac-*ac109*Rep). All of them carried the *polyhedrin* and *egfp* genes driven by the polyhedrin and p10 promoter, respectively. Bac-PHGFP contained an intact copy of *ac109*, Bac-*ac109*KO harbored a 670 bp-fragment of *ac109* replaced by a chloramphenicol cassette, and Bac-*ac109*Rep had *ac109* replaced as before but *ac109*, along with its own promoter, was inserted in the polyhedrin *locus* together with the *polyhedrin* and *egfp* genes. All constructs were verified by PCR (data not shown). Analyses of GFP fluorescence at 96 h post-transfection (hpt) after cell transfection with recombinant bacmids revealed that Bac-*ac109*KO gave rise to a single cell-infection phenotype, while Bac-PHGFP and Bac-*ac109*Rep produced virus that disseminated in the cultured cells (Fig. 1A, left panel); which is in accordance with previous reports. Infectious BV production was measured by titration of culture supernatants at various times after transfection. No differences were detected between Bac-PHGFP and Bac-*ac109*Rep. In contrast, no infectious BVs were detected in culture supernatants of cells transfected with Bac-*ac109*KO (Fig. 1B). To determine if the cells transfected with Bac-*ac109*KO produced budded viruses, we examined preparations of concentrated culture supernatants by immunoblotting and TEM. Western blot analysis with specific antibodies against the major nucleocapsid protein VP39 and the envelope glycoprotein GP64 revealed specific bands of both proteins (data not shown), suggesting that enveloped nucleocapsids were present. Identical preparations were further observed by TEM and budded viruses of normal appearance were detected (Fig. 1C). These results confirmed the presence of non-infectious virions produced from cells transfected with Bac-*ac109*KO. Altogether, these results confirm that although Ac109 is not required for budded virus production, this protein is involved in their infectivity. In addition, the results indicate that Ac109 is dispensable for very late gene expression, since *eGFP* driven by p10 promoter could be detected in cells transfected with Bac-*ac109*KO.

Light microscopy analysis showed that cells transfected with Bac-*ac109*Rep and Bac-PHGFP produced polyhedra of normal appearance. These polyhedra were observed 72 hpt in almost every cell of the monolayer. In turn, cells transfected with Bac-*ac109*KO displayed normal-looking polyhedra only in the originally transfected cells. Only half of the fluorescent cells, however, produced polyhedra, suggesting that the deletion of *ac109* affects the morphogenesis of normal occlusion bodies (Fig. 1D).

### Complementation Assays

Co-transfection assays were performed to evaluate if *ac109* deletion could be rescued in *trans*. Virus dissemination was observed when Bac-*ac109*KO was co-transfected with TOPOp109-109 or p_XXL_109; these plasmids contain the *ac109* gene under the regulation of its own late promoter (p109∶109) or of the very late polyhedrin promoter (p_XXL_:109). In contrast, when Bac-*ac109*KO was transfected along with pIB109 plasmid, in which the early promoter pOpIE2 drives the expression of *ac109*, eGFP fluorescence remained restricted to the originally transfected cells (Fig. 1A, right panel). In these experiments, the expression of Ac109 was confirmed by RT-PCR (data not shown).

To determine if Ac109 protein expressed in *trans* can rescue the infectivity of *ac109* knockout budded virus (*ac109*KO BVs), we infected Sf-9 cells previously transfected with pIB109 with *ac109*KO BVs. In these cells, eGFP fluorescence could not be observed (data not shown). The same result was obtained when Sf-9 cells were co-infected with wild type (wt) and *ac109*KO BVs. Therefore, *ac109* deletion could only be complemented in *trans* when Ac109 protein is expressed late or very late in the virus infection cycle. The presence of the Ac109 protein in the cytoplasm of non-infected cells or attached to co-infecting viruses failed to save the inability of *ac109*KO BVs to infect Sf-9 cells.

### Ac109 Localization in Virions and in Living Sf-9 Cells

**Figure 2:**
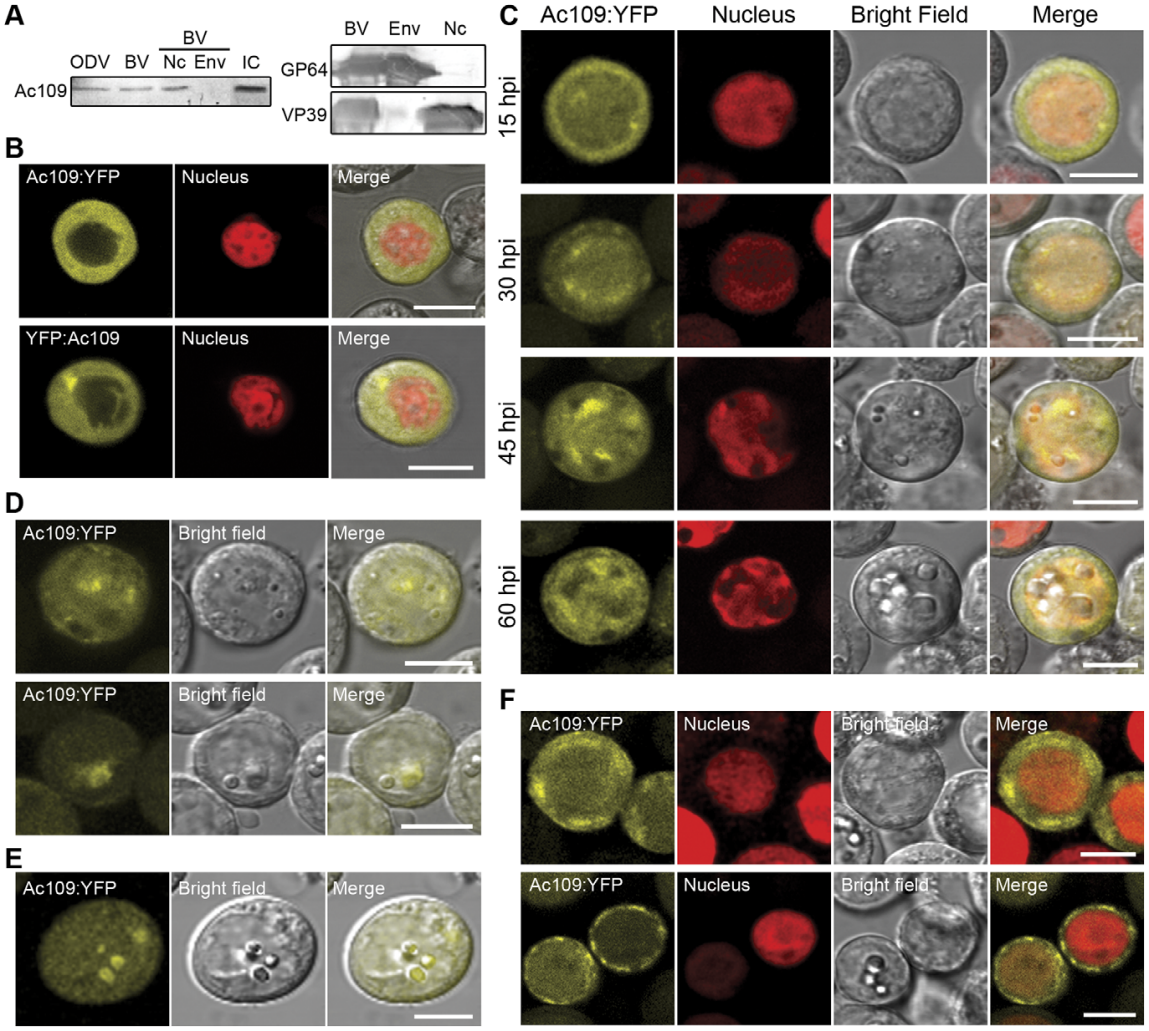
Ac109 localization in virions and in Sf-9 cells. (A) Western blot analysis of purified virions. Budded viruses (BVs), obtained from supernatants of Sf-9 cells infected with wild type (wt) *Ac*MNPV at a multiplicity of infection (MOI) of 0.1, were purified through a 25% w/v sucrose followed by a 25–65% sucrose gradient. Purified BVs were resuspended in PBS, and nucleocapsids and envelopes were separated using 1% IGEPAL and ultracentrifugation through a glycerol cushion. Occlusion derived viruses (ODVs) were obtained after alkaline treatment of polyhedra recovered from the infected Sf-9 cells. ODVs were purified through a discontinuous 30–65% sucrose gradient. Ac109, GP64 and VP39 proteins were detected from purified ODVs, BVs, BVs envelopes (Env) and nucleocapsid (Nc) fractions with a polyclonal serum anti Ac109-GST fusion protein, an anti-GP64 monoclonal antibody or an anti-VP39 monoclonal antibody. Sf-9 cells infected with wt *Ac*MNPV (IC) were used as a control of Ac109 detection. (B) Subcellular localization of Ac109 in living cells. Ac109 fused to YFP protein at either C or N-terminus (Ac109:YFP and YFP:Ac109) and transcribed from pOpIE2 promoter was detected in Sf-9.RNuc cells by confocal microscopy at 48 h post-transfection (hpt). (C) Kinetics of subcellular localization of Ac109 during baculovirus infection. Confocal microscopy of Sf-9.RNuc cells infected with Acppolp109-109Y virus at a MOI of 5 is shown at indicated h post-infection (hpi). Ac109 was fused to YFP at the C-terminal end (Ac109:YFP) and transcribed from the ac109 promoter. (D) Living insect cells infected with Acppolp109-109Y virus showing a heterogeneous Ac109:YFP accumulation around forming polyhedra in the nucleus. (E) Ac109:YFP fluorescence concentrated in the already formed polyhedra. (F) Cytoplasmic accumulations of Ac109:YFP in Sf-9.RNuc cells at different stages of viral infection. Bars represent 10 µm.

The localization of Ac109 in viral particles was then analyzed. Wt *Ac*MNPV ODVs and BVs were concentrated and purified from infected Sf-9 cells and supernatants of infected cells, respectively. Purified BVs were subsequently fractionated into envelopes and nucleocapsids. In these samples, Ac109 was immunodetected using a specific mouse polyclonal antiserum obtained from Balb/c mice inoculated with purified Ac109 protein fused to GST. Ac109 was revealed in both viral phenotypes (BVs and ODVs), and it was only detected in the nucleocapsid fraction of BVs. The effectiveness of fractionation was confirmed by Western blot using antibodies against VP39 and GP64;which are specific for nucleocapsid and envelope, respectively (Fig. 2A).

The subcellular localization of Ac109 when expressed alone or in the context of infection of living Sf-9 cells was tracked by confocal microscopy using translational fusions of Ac109 with YFP. Ac109 was fused to YFP to either its N or C-terminal end and transcribed driven by the constitutive pOpIE2 promoter in insect cells. The resulting plasmids pIB-Y-Ac109 and pIB-Ac109-Y were transfected in a polyclonal cell line (Sf-9.RNu) that expresses a red fluorescent nuclear protein (see Materials and Methods) to unequivocally differentiate cell cytoplasm and nucleus. Confocal analysis showed that, at 48–72 hpt, both YFP:Ac109 and Ac109:YFP were almost exclusively cytoplasmic (Fig. 2B).

In order to determine if Ac109 subcellular distribution depends on other factors present in baculovirus-infected cells, we produced the recombinant baculovirus Acppolp109-109Y. This virus carried the *polyhedrin* gene under the regulation of its own promoter and a second copy of *ac109* fused to the 5′ end of the YFP sequence in the *polyhedrin locus*. Sf-9 cells infected with Acppolp109-109Y were monitored from 5 to 72 h post-infection (hpi) and Ac109:YFP subcellular localization was monitored every 60 min (Fig. 2C). A weak Ac109:YFP fluorescence was first detected at 7 hpi, and it increased rapidly at 11–12 hpi (data not shown). During this period, the protein was detected mainly in the cytoplasm, and then, over the progression of the viral cycle (30 hpi), it entered the nucleus, clearly accumulating in the ring zone. Later, at approximately 45 hpi, the fluorescence was heterogeneously concentrated in defined regions of the nucleus. Very late in the viral infection cycle (60 hpi), Ac109:YFP fluorescence became weaker and more diffuse in the nucleus and cytoplasm. In some cells, clusters of fluorescent protein could be observed around forming polyhedra (Fig. 2D), and later in other cells these clusters coincided with the already formed occlusion bodies (Fig. 2E). In addition, cytoplasmic accumulations were observed at different times post-infection in cells where Ac109:YFP was strongly expressed (Fig. 2F).

### Effect of ac109 Deletion on BVs Entry to the Cell and Cell Nucleus

**Figure 3:**
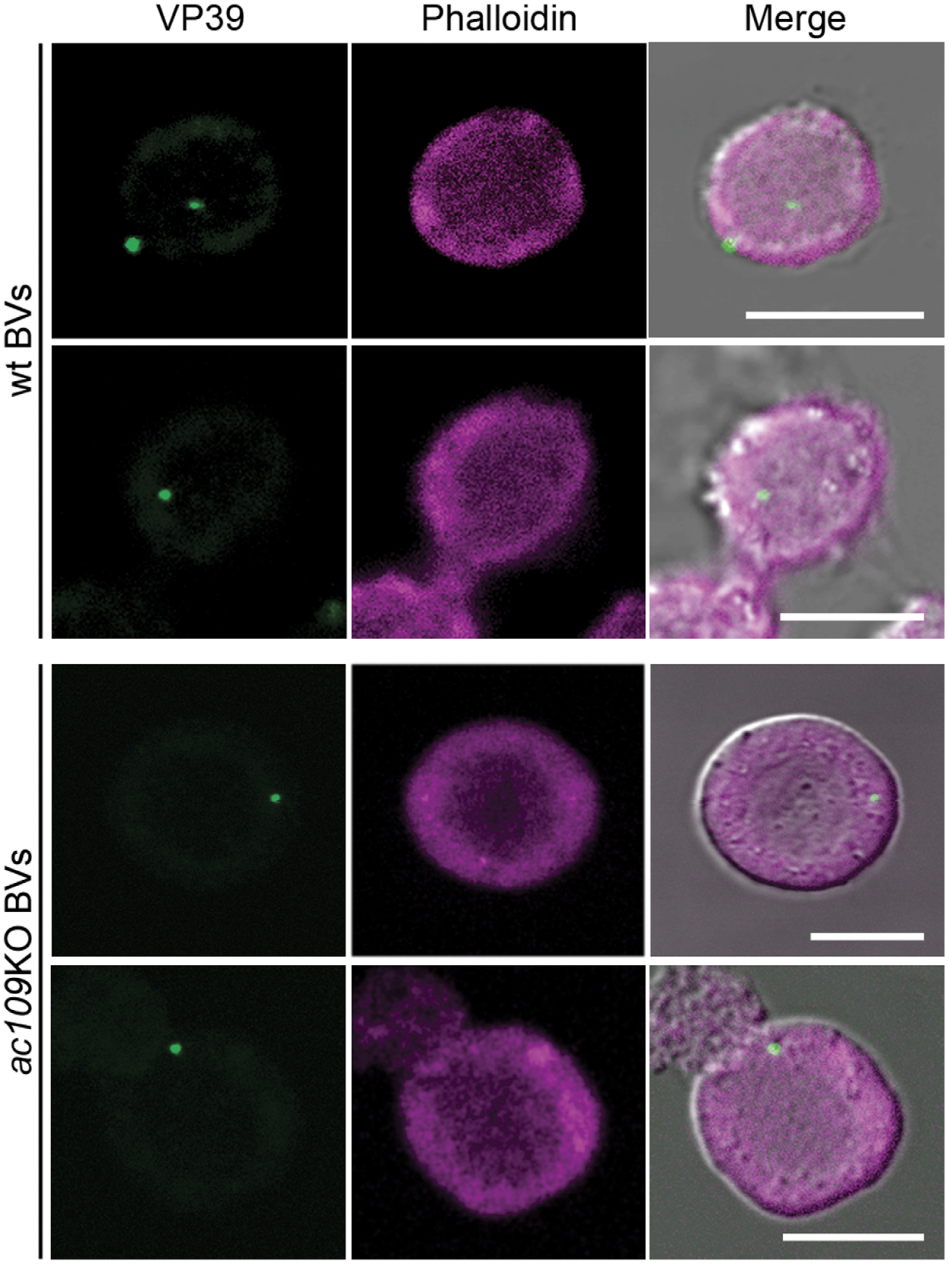
Detection of the entrance of *ac109* knockout-baculovirus in insect cells. Immunofluorescence of insect cells infected with wild type (wt) *Ac*MNPV or with *ac109*KO BVs. Sf-9 cells were incubated with wt *Ac*MNPV at a multiplicity of infection of 0.5 or supernatants of Bac-*ac109*KO transfected cells for 45 min at 4°C. Then, cells were attached on culture dishes and incubated for 2 h at 27°C. Fixed cells were treated with an anti-VP39 monoclonal antibody and an Alexa Fluor® 488 conjugated anti-mouse antibody. Next, cells were stained with Alexa Fluor® 633 phalloidin and observed by confocal microscopy. Bars represent 10 µm.

With the objective of identifying the step affected in *ac109*KO viral cycle that impairs the virus spread, we examined *ac109*KO BVs infection capacity in Sf-9 cells by immunofluorescence and confocal microscopy. Wt and *ac109*KO BVs were adsorbed in Sf-9 cells and then incubated for 2 h at 27°C. Cells were then fixed and *ac109*KO BVs localization was followed by detecting the nucleocapsid protein VP39. As expected, viral particles were detected in both the cytoplasm and nucleus of cells infected with wt virus at 2 hpi. In contrast, VP39 could be detected exclusively in the cytoplasm of cells infected with *ac109*KO BVs (Fig. 3). These results demonstrated that *ac109*KO BVs were able to adsorb and enter insect cells. However, they were impaired in their capacity to reach the cell nucleus, therefore, remaining in the cytoplasm.

### Effect of ac109 Deletion in ODV Occlusion and Infectivity

**Figure 4:**
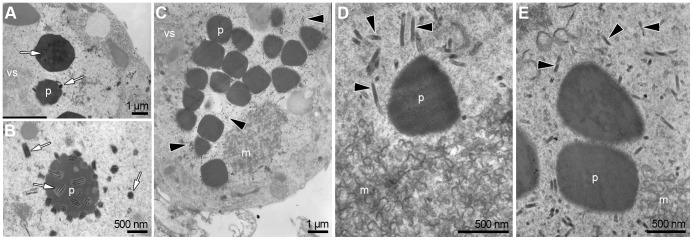
TEM analysis of the morphogenesis of occlusion bodies in the absence of Ac109. Representative images of Sf-9 cells transfected with (A) Bac-PHGFP (B) Bac-*ac109*Rep or (C–E) Bac-*ac109*KO bacmids. White arrows indicate enveloped nucleocapsids present in cells transfected with Bac-PHGFP and Bac-*ac109*Rep. Black arrows show naked nucleocapsids accumulating around empty polyhedra in Bac-*ac109*KO transfected cells. vs: virogenic stroma, p: polyhedron, m: microvesicles.

Finally, we investigated the effect of *ac109* deletion in the occlusion process. Sf-9 cells were transfected with Bac-PHGFP, Bac-*ac109*KO and Bac-*ac109*Rep and at 96 hpt cells were fixed and observed by TEM. Typical changes proper of baculovirus infection such as the presence of virogenic stroma, rod-shaped nucleocapsids aligned with inner nuclear membranes, virions enveloped in groups and embedded in crystalline structures were observed in the nuclei of cells transfected with Bac-PHGFP and Bac-a*c109*Rep (Figs. 4A and B). An extensive scan of the sections revealed that cells transfected with Bac-*ac109*KO also showed some of these elements, including the presence of virogenic stroma. Moreover, in about half of the cells with signs of infection, polyhedra could be found. Interestingly, all of the observed polyhedra did not contain occluded virions. Moreover, they were surrounded by numerous bundles of non-enveloped and rod-shaped nucleocapsids (Figs. 4C, D and E, black arrowheads). Microvesicles, probably synthesized *de novo*, accumulated near non-aligned nucleocapsids (Figs. 4C, D and E). In accordance, *Rachiplusia nu* larvae fed with a diet contaminated with polyhedra derived from *ac109*KO-infected cells did not show any symptom of infection (data not shown). Overall, these results clearly demonstrated that *ac109* deletion totally abolished the normal morphogenesis of ODVs, although it failed to abrogate the production of polyhedra.

## Discussion

In this study, we studied the role of *ac109*, one of the *Ac*MNPV 31 core genes [9] of unknown function. Previous studies have demonstrated that Ac109 protein is essential for virus dissemination in cultured cells and is not required for viral DNA replication [7], [8]. Nevertheless, its participation in the production of budded virus and in the morphogenesis of occlusion bodies has not been clearly defined. In this study, we generated an *ac109* knockout baculovirus and established that Ac109 is involved in *Ac*MNPV infectivity, impairing the ability of BVs to enter the nucleus and the ability of ODVs occlusion in polyhedra.

Fluorescence microscopy analysis of cells transfected with an *ac109* knockout bacmid not only confirmed the failure of the mutant to disperse the infection beyond the transfected cells, but also demonstrated that in the absence of Ac109 very late transcription is not affected, since *egfp* under p10 regulation was normally expressed. Next, complementation studies revealed that a productive infection was achieved when Ac109 was expressed in *cis* or in *trans* at a suitable timing of the viral infection. On the contrary, if *ac109* was transcribed in cells early in the viral cycle, infectivity of *ac109* knockout bacmid was not rescued. Therefore, the Ac109 available when the ac109 gene is placed under the control of the early pOpIE2 promoter may not be sufficient for Ac109 to adequately fulfil its function owing to the shut down of early genes induced by the infection. The analysis of Ac109 fused to YFP in living cells by confocal microscopy revealed that in the absence of infection, Ac109 is a cytoplasmic protein. During the course of the infection, however, Ac109 is directed to the nucleus and accumulates first in the ring zone and later in other nuclear regions; therefore, confirming previous findings using immunofluorescence in fixed cells [8]. The fact that Ac109 localized in the cytoplasm when constitutively expressed in insect cells reveals that there should be at least a viral protein or a viral-induced cellular factor involved in its transport to the nucleus. Ac109 has been previously shown to be present in ODVs and BVs using proteomic approaches [10], [11]. However, the strategy employed failed to distinguish between actual integral structural components and proteins non-specifically trapped during virion assembly [9]. In addition, Fang et al. have detected an HA-tagged version of Ac109 at similar levels in both the BVs nucleocapsid and envelope fractions [8]. Our results are in agreement with the localization of Ac109 in both ODV and BVs phenotypes. However, in contrast to Fang et al.’s results, we detected Ac109 in the nucleocapsid fraction of BVs but not in their envelopes. Although the sensitivity of the Western blot assays could be different, our results clearly revealed at least a pronounced differential presence of Ac109 in nucleocapsids. Alternatively, the presence of the HA tag could negatively affect the association between Ac109 and nucleocapsids giving rise to an Ac109 contamination of the envelope fraction.

Previous studies differ as to the ability of *ac109* knockout virus to trigger nucleocapsid assembly and BVs production [7], [8]. In this report, enveloped viral particles were recovered from supernatants of *ac109* knockout-infected cells. Moreover, the fact that the *ac109*KO infectivity could not be recovered when Ac109 was expressed *in trans* in cells prior to infection or when it was present during infection with wt budded viruses suggests a function for Ac109 in the first steps of the viral infection cycle as an integral part of the virion structure. Ac109 knockout BVs were able to enter the cells, as revealed by the presence of nucleocapsids in the cytoplasm of newly infected Sf-9 cells. However, it was not possible to detect nucleocapsids in the cells’ nuclei, which indicates either a failure in the escape of the ac109 knockout virions from the endosome or in their nuclear import. Since the major envelope glycoprotein GP64 mediates low-pH-triggered membrane fusion [12] and our results showed that it is present in *ac109* knockout viral particles and that the cell entrance is not affected in this mutants, it is unlikely that the absence of Ac109 impairs the fusion of viral and endosome membranes. *Ac*MNPV nucleocapsids are released from endosomes and undergo intracellular motility to reach the nucleus by a process driven by actin polymerization [13], [14], and, finally, *Ac*MNPV/the protein is relocalized to the nucleus throughout the progress of infection. These processes involve several host and viral factors such as P78/83, Ac42, Ac102 [15] and Arp2/3 complexes [16], [17]. It may be hypothesized that Ac109 is required indirectly to maintain the adequate nucleocapsid architecture in its interaction with cellular actin or directly participating in this interaction. Alternatively, the transport through the nuclear pores could be affected. By using *Ac*MNPV capsids and *Xenopus* oocytes cell system, Au and Panté [18] have recently demonstrated that intact nucleocapsids enter into the nucleus through nuclear pores providing evidence of an interaction of the conical end of the capsids with cytoplasmic filaments of the nuclear pores. If this mechanism is specific and necessary for nucleocapsid entrance into nucleus, it is probable that some capsid proteins such as Ac109 are involved in the process.

Regarding the impact of *ac109* deletion in the occlusion process, previous reports have also arrived to discordant conclusions when analyzing the presence of polyhedra in cells by light microscopy. Whereas Fang et al. found occlusion bodies of normal appearance [8], Lin et al. did not [7]. In this respect, our findings (using *ac109* knockout bacmid) resulted in the observation of polyhedra in about half of the transfected cells under light microscopy. Under electron microscopy, however, we observed that these polyhedra are empty, and that nucleocapsids around the polyhedra are non-enveloped. Deletions of other genes and mutants in polyhedrin protein have been described to produce empty polyhedra, indicating that occlusion is not a requisite for polyhedrin polymerization [19]–[23], although the crystals fail to form *in vitro* or in uninfected cells; which implies that polyhedron morphogenesis depends on the presence of viral products. ODV morphogenesis is a complex process, as it requires the coordinate action of several genes. In addition, the disturbance of any of these factors produces disruption of the mechanism underlying virion envelopment and subsequent occlusion. Several ODV proteins have been described to participate in this last stage of infection; disruption of either *ac142* [22], *p48* [*23*], *ac53*, [20], *ac76*, [24], *38K* [25] or *ac92* [26] affects occlusion of virions in the cell nucleus. While disruption of *38K* or *ac53* leads to defects in nucleocapsid assembly, knockouts of *ac142*, *ac76 ac92* or *p48* interfere with nucleocapsid envelopment, as we observed for *ac109* knockout in this study. Interestingly, protein-protein interaction has been reported for homologs of the *ac109* and *ac142* genes in *Helicoverpa armigera* nucleopolyhedrovirus using the Gal4 yeast two-hybrid system [27]. Analysis of the subcellular distribution of Ac109, which showed protein accumulation in the ring zone and around forming polyhedral, could also correlate with an active role of Ac109 in the morphogenesis of occlusion bodies. Electronic microscopy of *ac109*KO-transfected cells also revealed abnormal accumulation of microvesicles in the nuclear periphery. These microvesicles are derived from invaginations of the inner nuclear membrane or are formed by *de novo* morphogenesis, and their production is independent from the envelopment process [4]. Our results indicate that microvesicle synthesis is not affected by the absence of Ac109. Therefore, Ac109 might be implicated in the interaction between nucleocapsids and nuclear membranes or could be required for an adequate architecture of nucleocapsids that allows this interaction.

In sum, our findings suggest that the multiple effects observed in the absence of Ac109 are probably due to an alteration in the architecture of virions; which affects the normal transport or entrance of BVs into the nucleus and the envelopment and occlusion of virions in polyhedra. Additional information about Ac109 interactions with other viral or cellular proteins could contribute to explain the precise role of Ac109 in these processes.

## Materials and Methods

### Cells and Viruses

Sf-9 insect cell line, the clonal isolate 9 from IPLB-Sf21-AE cells, derived from the fall armyworm *Spodoptera frugiperda*, was purchased from Invitrogen and maintained at 27°C in Sf-900 ™ II serum free insect medium (Invitrogen). Wt *Ac*MNPV was obtained from Pharmingen (BD Biosciences). Wt and recombinant baculoviruses were propagated in Sf-9 cells.

For the establishment of the Sf-9.RNu cell line, 1×10^6^ cells growing in 35 mm-diameter culture dishes were transfected with 1 µg of plasmid pIB-LMNA-R [28] using Cellfectin II® reagent (Invitrogen). Recombinant cellular clones were selected using blasticidin, at concentration of 60 µg/ml. Confluent monolayers were subsequently subcultured in larger plates, while blasticidin concentration was reduced to up to 20 µg/ml, which was the concentration used to maintain cell line.

Bacmid and plasmid transfections were performed with 1 µg of DNA per 1×10^6 ^Sf-9 cells using Cellfectin II® transfection reagent according to the manufacturer’s instructions.

### Generation of Recombinant Plasmids, Bacmids and Viruses

#### Bac-PHGFP, Bac-ac109KO and Bac-ac109Rep

A chloramphenicol resistance cassette was amplified using following primers: Cn109F 5′ AACATAGACAGTCGATCGCCAAAAAAAACAGCGTCCGCCACCATAGTCTA TTTAAGGGCACCAATAACTG 3′ and Cn109R 5′ TTCTTGCAAAACAGCCGAATTTTTTTGTAGGGCTCTGCTTTATTCGGCGC TTCCTGTGCGACGGTTAC 3′ and the plasmid pBeloBac11 [29] as a template. These primers contain 50 bp homologous to the 5′ and 3′ ends of the coding region of *ac109* gene (underlined). The linear PCR fragment was gel-purified and electroporated in *E. coli* BJ5183 cells [30], [31] which already contained bMON1472 bacmid, previously obtained from *E. coli* DH10Bac strain (Invitrogen). Cells were allowed to recover during 4 h at 37°C in SOC medium (Invitrogen, USA) and plated in LB agar containing 100 µg/ml chloramphenicol and 50 µg/ml kanamycin. Resistant colonies were selected and the presence of the bacmid *ac109*KO was confirmed by PCR. Primers 1584 [8] and CmF 5′ GTAACCGTCGCACAGGAA 3′ were used to verify the correct insertion of the chloramphenicol cassette in the *ac109* locus; primers 1584 and 109F2F 5′ GGTGAAGATATTGTCGTTGAAACGC 3′ amplified *ac109* gene from non-deleted genomes.

pFBD-PHGFP and pFBD-PHGFPp109-109 transfer vectors were constructed from pFastBacDual plasmid (Invitrogen) inserting the *polyhedrin* gene under its own promoter regulation, *egfp* downstream of p10 promoter and *ac109* with its native promoter. For the construction of the intermediate vector pFBDPH, The *polyhedrin* gene was obtained from TOPO-POL vector [32] digested with EcoRI, and cloned into pFastBacDual digested with the same enzyme. The 716 bp *eGFP* coding sequence was amplified using oligonucleotides GFP-for 5′ GCTCGAGATGGTGAGCAAGGGCGAG 3′ and GFP-rev 5′ GTCTCGAGTTATTGTACAGCTCGTCCATGC 3′, both indicated with underlined XhoI restriction enzyme sites. *egfp* gene was cloned into pGEMT-easy vector (Promega) and subcloned into pFBDPH plasmid using the XhoI site to obtain pFBD-PHGFP vector. *ac109* and its promoter were amplified from DNA extracted from cells infected with wt *Ac*MNPV using the following primers: 109Prom 5′ TTATCATGACCATTACAATTACGTGCCCG 3′ with a BspHI enzyme site (underlined) and Orf109R 5′CTACAAATAATAGTTGTACTTGACGG 3′, and cloned into pCR®2.1-TOPO® (Invitrogen) vector to obtain TOPOp109-109. This plasmid was digested with PvuII and SpHI enzymes and cloned into pFBD-PHGFP digested with the same enzymes, to obtain pFBD-PHGFPp109-109. bMON1472.109KO bacmid and pMON7124 plasmid were co-electroporated into *E. coli* DH10 strain, and transformants were selected in LB agar with chloramphenicol, kanamycin and 10 µg/ml tetracycline. Finally, Bac-*ac109*KO and Bac-*ac109*Rep were obtained by Tn7-mediated transposition between bMON1472.109KO and pFBD-PHGFP or pFBD-PHGFPp109-109, as previously described [33]. Bac-PHGFP was obtained using Bac-to-Bac system (Invitrogen) with pFBD-PHGFP as transfer vector, according to the manufacturer’s instructions.

The bacmids were purified from bacteria using Large-Construct purification kit (QIAGEN) following the manufacturer’s instructions.

#### p_XXL_109, pIB109 and TOPOp109-109 plasmids

Complementation assays were performed using p_XXL_109, pIB109 and TOPOp109-109 plasmids. The construction of TOPOp109-109 was described in the previous section. *ac109* sequence was PCR amplified to obtain p_XXL_109 and pIB109 from DNA extracted from cells infected with wt *Ac*MNPV using the following primers: Orf109F: 5′ ATGGAGTGCCCGTTTCAGC 3′ and Orf109R. For the construction of the pIB109 vector, *ac109* was cloned into pCR®8/GW/TOPO® (Invitrogen) and subcloned into pIB-V5/His-DEST (Invitrogen) by recombination using the LR Clonase II enzyme mix (Invitrogen), according to manufacturer’s protocols. As for p_XXL_109 plasmid, *ac109* was cloned into pCR®2.1-TOPO® vector, excised with SpeI and XhoI enzymes and subcloned into p_XXL_CAT vector [32].

#### pIB-Y-109 and pIB-109-Y plasmids

To obtain Ac109 N-terminal or C-terminal fluorescence fusions, we recombined TOPO.109N entry vector containing the *ac109* coding sequence without the ATG initiation codon or TOPO.109C entry vector containing the *ac109* coding sequence lacking the STOP codon into pIB-YW or pIB-WY destination vectors [28] using the Gateway system (Invitrogen), giving rise to pIB-Y-109 and pIB-109-Y vectors respectively.

### Acppolp109-109Y

The Bac-to-bac system was used to obtain a baculovirus carrying a second copy of *ac109* fused to YFP downstream of its own promoter. Transfer vector pFBpPOLp109-109Y was constructed replacing in pFasBacDual vector polyhedrin and p10 promoters by *polyhedrin* gene and an upstream regulatory region of 323 bp and a C-terminal fusion of *ac109* with *yfp* gene downstream of *ac109* promoter. *polyhedrin* gene and its promoter were PCR amplified with primers PH-up 5′ GCCGGCATAGTACGC 3′ and POLSTOP [32] and cloned into pGEM-Teasy intermediate vector giving rise to pGEMpPOL. *ac109* promoter sequence and *ac109* gene without the STOP codon were amplified by PCR and cloned in pCR®8/GW/TOPO® to obtain TOPO.p109-109C entry vector, which was recombined into pIB-WY destination vector [28]. p109-109Y sequence was amplified with 109Pr and GFP-rev primers and cloned into pCR®2.1-TOPO® vector. The sequence of *polyhedrin* and its promoter and p109-109Y sequence were obtained digesting pGEMpPOL or TOPOp109-109Y with NcoI and NsiI or BspHI and NotI enzymes, respectively. Both DNA fragments were cloned into pFasBacDual vector digested with NsiI and NotI enzymes. Acppolp109-109Y baculovirus was obtained after transfection of Sf-9 cells with pFBpPOLp109-109Y, amplified and titrated by the end point dilution method.

All transfer and intermediate vectors were sequenced to confirm their identity.

### Quantification of Infectious BVs

Sf-9 cells (1×10^6^) were seeded in 35 mm-diameter dishes and transfected with BacPHGFP, Bac-*ac109*Rep or Bac-*ac109*KO. At 18, 24, 44, 68 and 92 h post-transfection (hpt) cell’s supernatants were collected, clarified and tittered in triplicate in Sf-9 cells by the end point dilution method [34].

### Purification of BVs and ODVs

To purify BVs, we transfected Sf-9 cell monolayers with Bac-*ac109*KO bacmid or infected with wt *Ac*MNPV at a multiplicity of infection (MOI) of 0.1. Four days after transfection or infection, supernatants were harvested and centrifuged through a 25% w/v sucrose cushion at 80,000×g for 75 min. The pellet containing *ac109*KO BVs was resuspended in phosphate buffer saline (PBS) and analyzed by Western blot or by TEM. Wt BVs were loaded onto a 25–60% sucrose gradient and centrifuged at 96,000×g for 3 h at 4°C. Next, the band containing viruses was harvested, and diluted in PBS, then purified BVs were concentrated by centrifugation at 80,000×g for 1 h. Pellets were resuspended in PBS at a final concentration of 1×10^11^ pfu/ml. Envelopes and nucleocapsids were fractionated as previously described, [35] with the following modifications: purified BVs were treated with 1% IGEPAL overnight at 4°C, and then loaded in a 1 ml glycerol cushion and centrifuged at 100,000×g for 1 h at 4°C. The pellet containing the nucleocapsid fraction was resuspended in 0.1X TE and the supernantant containing the envelopes was precipitated with 10% trichloroacetic acid for 1 h at 4°C, centrifuged at 16,000×g for 30 min at 4°C and resuspended in 0.1X TE.

For ODVs purification, once the culture supernatant was harvested to obtain BVs, fresh culture medium was added and the cells were incubated at 27°C. Seven days post-infection the supernatant was collected, clarified and discarded. Cells and polyhedra were treated with 0.5% SDS, 0.5 M NaCl, washed with bidestilated water and polyhedrin was dissolved with 0.1 M Na_2_CO_3_ 30 min at room temperature. Then, the sample was loaded onto a discontinuous 30–65% sucrose gradient, centrifuged at 96,000×g for 90 min at 4°C and the band was collected and diluted in PBS. Purified ODVs were pellet by ultracentrifugation at 80,000×g for 1 h and diluted in a small volume of 0.1X TE.

### Western Blot Analyses

Purified BVs or fractions containing nucleocapsids or envelopes were boiled for 5 min in disruption buffer (120 mM Tris–HCl pH 6.8, 4% SDS, 0.02% bromophenol blue, 1.4 M ß-mercaptoethanol, 20% glycerol) and proteins were resolved in 12% acrylamide-bisacrylamide (30∶0.8) SDS-PAGE minigels (Bio-Rad). Proteins were blotted onto nitrocellulose membranes and the blots were probed with a mouse polyclonal antiserum anti-Ac109GST (1∶50), a mouse monoclonal anti-VP39 (1∶2,000) [36] or a mouse monoclonal anti-GP64 (AcV5, diluted 1∶500) [37] primary antibodies followed by an alkaline phosphatase-conjugated anti-mouse antibody (1∶15,000) (SIGMA). For the anti-GSTAc109 serum obtaining, *ac109* was amplified by PCR using primers: Orf109FBam: 5′ CGGATCCGAGTGCCCGTTTCAGATTC 3′ and Orf109RXho 5′ CCTCGAGCTACAAATAATAGTTGTAC 3′ with *a* BamHI or XhoI enzyme site, respectively (underlined), and then it was cloned into pGEM-Teasy intermediate vector. pGEM109 was digested with *BamH*I and *Xho*I and cloned in pGEX-6P-1 vector (GE Healthcare) in frame with C-terminal coding sequence of GST protein. *E. coli* BL21 strain containing pGEX-109 were grown at OD_600_∶1 and induced with 0.1 mM IPTG at 23°C for 1 h 30 min. Bacteria were pelleted, resuspended in 1 mg/ml lyzozyme in PBS and sonicated. Next, Triton was added to a final concentration of 1% and the sample was incubated for 30 min in ice. The fusion protein was purified using Glutation Sepharose ™ 4B (GE Healthcare) resin following the manufactureŕs instructions and antisererum was obtained immunizing Balb/c mice using Freund’s incomplete adjuvant. The specificity of anti-GSTAc109 serum was verified by Western blot.

### Complementation Assays

To evaluate *trans* complementation of Bac-*ac109*KO, we co-transfected monolayers of 8×10^4 ^Sf-9 cells with Bac-*ac109*KO and TOPOp109-109, pIB109 or p_XXL_109 plasmids. One×10^6 ^Sf-9 cells were transfected with Bac-*ac109*KO to determine *trans* complementation of *ac109*KO BVs. At 4 days post-tranfection (dpt) supernatants were collected, centrifuged to remove debris and used to infect 1×10^5 ^Sf-9 cells that were transfected with pIB109 3 days before. Culture supernatants containing *ac109*KO BVs were also used to co-infect 1×10^5 ^Sf-9 cells with wt *Ac*MNPV at MOI of 1. eGFP fluorescence was monitored from 24 to 96 hpt.

### Fluorescence Live Imaging

Sf-9 cells were transfected with each bacmid (Bac-*ac109*KO, Bac-PHGFP or Bac-*ac109*Rep) or co-transfected with Bac109KO and p_XXL_109, pIB109 or TOPOp109-109 and light and fluorescence images were captured using a Leica TCS-SP5 (Leica Microsystems GmbH, Wetzlar, Germany) spectral laser confocal microscope in epifluorescence mode with a mercury lamp and the I3 filter.

Cells transfected with pIB-Y-109 or pIB-109-Y were used for *in vivo* fluorescence imaging at 48–72 hpt in the same spectral laser confocal microscope using a 63× objective (HCX PL APO CS 63.0×1.20 WATER UV). To determine Ac109-YFP subcellular localization along the viral infection, we infected cells with Acppolp109-109Y virus at a MOI of 5 and fluorescence images were collected every 60 min from 5 to 72 hpi using a 40× oil objective (HCX PL APO CS 40.0×1.25 OIL UV). The 514 nm line of the Argon laser was used for YFP excitation and the 543 nm line from the HeNe laser was employed for mCherry excitation. Scanning was performed in sequential mode to eliminate signal bleed-through, and fluorescence emission was detected with the following channel settings: 525–600 nm for YFP and 610–670 nm for mCherry. The microscope power settings, detectors gain and scanning speed were adjusted to optimize contrast and resolution for each individual image.

### Immunofluorescence

2×10^4^ Sf-9 cells were infected in suspension with 4 ml of culture supernatants containing *ac109*KO BVs, or with wt *Ac*MNPV BVs at a MOI of 0.5. Viral adsorption was performed for 45 min at 4°C; next, cells were pellet, washed once with culture medium and seeded into Lab-Tek Chambered coverglass dishes (Nunc). Cells were incubated at 27°C for 2 hpi,washed with PBS, fixed in 4% paraformaldehyde (pH 7 in PBS) for 15 min, washed twice with PBS-T (PBS-0.05% Tween) and permeabilized with 0.2% Triton X-100 for 30 min. Cells were subsequently washed twice with PBS-T before treating with 1% Blocking Reagent (Roche) in PBS for 1 h. Next, cells were incubated with a mouse monoclonal anti-VP39 antibody (1∶25) [36] in blocking reagent for 90 min and washed twice with PBS-T to remove nonspecifically bound antibody. Cells were washed twice with PBS-T and incubated with an Alexa Fluor® 488 conjugated anti-mouse antibody (1∶800) (Molecular Probes) for 1 h. Samples were washed with PBS-T and incubated with 3 units of Alexa Fluor® 633 phalloidin (Molecular Probes) in 1% Bovine Serum Albumin (BSA) for 20 min at room temperature. Finally, cells were washed with distilled water, and mounted in ProLong Gold antifade reagent (Invitrogen). Samples were curated for 24 h at 4°C in the dark, and then visualized in a Leica TCS-SP5 spectral laser confocal microscope. Phalloidin visualization was performed by excitation with the 633 nm line of the HeNe laser and detection of fluorescence emission in the 650–750 nm spectral range.

### Electron Microscopy

For electron microscopy of *ac109*KO BVs, particles were bound to copper grids. To analyze transfected cells, we transfected monolayers of 3×10^7^ Sf-9 cells with Bac-*ac109*KO, Bac-PHGFP or Bac-*ac109*Rep and 5 dpt were fixed with 2.5% glutaraldehyde, post-fixed in 2% osmium tetroxide and packaged in agar. Samples were dehydrated using ethanol at ascending concentrations (50%, 70%, 96%, 100%) and acetone. The pre-inclusion in resin was made using one volume of acetone and one volume of Spurr resin. Blocks were then carved and sliced with an ultramicrotome to obtain section pieces of about 60–90 nm which were collected on copper grids. Samples were contrasted with heavy salts (coloration of uranyl-Reynold’s) and visualized in a TEM JEOL 1200EX-II microscope.
